# Seasonal changes in infection with trematode species utilizing jellyfish as hosts: evidence of transmission to definitive host fish via medusivory

**DOI:** 10.1051/parasite/2016016

**Published:** 2016-04-07

**Authors:** Yusuke Kondo, Susumu Ohtsuka, Takeshi Hirabayashi, Shoma Okada, Nanako O. Ogawa, Naohiko Ohkouchi, Takeshi Shimazu, Jun Nishikawa

**Affiliations:** 1 Takehara Marine Science Station, Seotuchi Field Science Center, Graduate School of Biosphere Science, Hiroshima University 5-8-1 Minato-machi Takehara Hiroshima 725-0024 Japan; 2 Institute of Biogeosciences, Japan Agency for Marine-Earth Science and Technology 2-15 Natsushima-cho Yokosuka 237-0061 Japan; 3 10486-2 Hotaka-Ariake Azumino Nagano 399-8301 Japan; 4 School of Marine Science and Technology, Tokai University, 3-20-1, Orido, Shimizu-ku, Shizuoka-shi Shizuoka 424-8610 Japan

**Keywords:** trematodes, scyphozoans, intermediate host, paratenic host, medusivory, trophic relationships

## Abstract

In the Seto Inland Sea of western Japan, metacercariae of three species of trematodes, *Lepotrema clavatum* Ozaki, 1932, *Cephalolepidapedon saba* Yamaguti, 1970, and *Opechona olssoni* (Yamaguti, 1934), were found in the mesoglea of the jellyfish *Aurelia aurita* s.l., *Chrysaora pacifica*, and *Cyanea nozakii*. Moreover, these jellyfish frequently harbored juveniles of the fish species *Psenopsis anomala*, *Thamnaconus modestus*, and *Trachurus japonicus.* The former two fish species are well-known medusivores. We investigated seasonal changes in the prevalence and intensity of these metacercariae in their host jellyfish from March 2010 to September 2012 and presumed that infection by the trematodes of the definitive host fish occurs through these associations. The mean intensity of metacercariae in *A. aurita* s.l. clearly showed seasonality, being consistently high in June of each year. The intensity of metacercariae in *C. nozakii* was highest among all jellyfish hosts and appeared to be enhanced by medusivory of this second intermediate, and/or paratenic host. Trophic interactions between jellyfish and associated fish were verified using both gut content and stable isotope analyses. The detection of trematodes and nematocysts in the guts of *P. anomala* and *T. modestus* juveniles, in addition to stable isotope analysis, suggests that transmission of the parasites occurs via prey-predator relationships. In addition, the stable isotope analysis also suggested that *P. anomala* is more nutritionally dependent on jellyfish than *Th. modestus* and *Tr. japonicus*.

## Abstract (fr)

Dans la mer intérieure de Seto à l’Ouest du Japon, les métacercaires de trois espèces de trématodes, *Lepotrema clavatum* Ozaki, 1932, *Cephalolepidapedon saba* Yamaguti, 1970 et *Opechona olssoni* (Yamaguti, 1934), ont été trouvées dans la mésoglée des méduses *Aurelia aurita* s.l., *Chrysaora pacifica* et *Cyanea nozakii*. De plus, ces méduses hébergeaient fréquemment des juvéniles des espèces de poissons *Psenopsis anomala*, *Thamnaconus modestus* et *Trachurus japonicus.* Les deux premières espèces de poissons sont connues comme médusivores. Nous avons étudié les variations saisonnières de prévalence et d’intensité de ces métacercaires dans leurs méduses hôtes de mars 2010 à septembre 2012 et présumé que l’infection des poissons hôtes définitifs par les trématodes se produit à travers ces associations. L’intensité moyenne des métacercaires chez *A. aurita* s.l. a clairement montré une saisonnalité, étant toujours élevée en juin de chaque année. L’intensité des métacercaires chez *C. nozakii* était la plus élevée parmi toutes les méduses hôtes et semblait être renforcée par médusivorie de ce second hôte intermédiaire, et/ou par un hôte paraténique. Les interactions trophiques entre les méduses et les poissons associés ont été vérifiées en utilisant à la fois le contenu de l’intestin et des analyses des isotopes stables. La détection de trématodes et de nématocystes dans les intestins de juvéniles de *P. anomala* et de *Th. modestus,* en plus de l’analyse des isotopes stables, suggère que la transmission des parasites se produit par l’intermédiaire de relations proies-prédateurs. En outre, l’analyse des isotopes stables a également suggéré que *P. anomala* est plus dépendant des méduses pour sa nutrition que *Th. modestus* et *Tr. japonicus*.

## Introduction

Jellyfish not only play an important role as predators in the marine ecosystem, but they also function as prey and hosts for a wide variety of organisms [1, 2, 38, 39, 41]. Interactions between jellyfish and fish have long been known and have been comprehensively reviewed by many authors [1, 3, 17, 19, 24, 41, 51, 52]. Jellyfish are utilized by fish for school formation, food collection, and prey [29, 30]. In addition, endoparasitic helminths are transmitted from intermediate host jellyfish to definitive host fish via predation [25]. Some digenean trematodes are known to use cnidarians as their second intermediate hosts [15, 28, 33] and/or paratenic hosts [20, 47–49]. Medusivorous fish become infected by trematodes through predation of infected jellyfish and act as definitive hosts [7, 39].

In the Seto Inland Sea of western Japan, some large scyphozoan jellyfish, such as the moon jellyfish *Aurelia aurita* (Linnaeus, 1758) s.l., Japanese sea nettle *Chrysaora pacifica* (Goette, 1836), and ghost jellyfish *Cyanea nozakii* Kishinouye, 1891, are infected by the metacercariae of trematodes [39]. Unencysted metacercariae of three species, *Lepotrema clavatum* Ozaki, 1932, *Cephalolepidapedon saba* Yamaguti, 1970, and *Opechona olssoni* (Yamaguti, 1934), have been found in the mesoglea of *A. aurita* s.l. [39]. In addition, these jellyfish are usually accompanied by other symbionts, including juveniles of the Japanese butterfish *Psenopsis anomala* (Temminck and Schlegel, 1844), black scraper *Thamnaconus modestus* (Günther, 1877), and Japanese jack mackerel *Trachurus japonicus* (Temminck and Schlegel, 1844) [30, 39].

The present study aimed to investigate seasonal changes in the prevalence and intensity of metacercariae of three trematode species in host jellyfish in the Seto Inland Sea from March 2010 to September 2012, and to clarify transmission of these trematodes to their definitive host fish.

## Materials and methods

### Seasonal changes in prevalence and intensity of metacercariae in jellyfish

**Figure 1.**
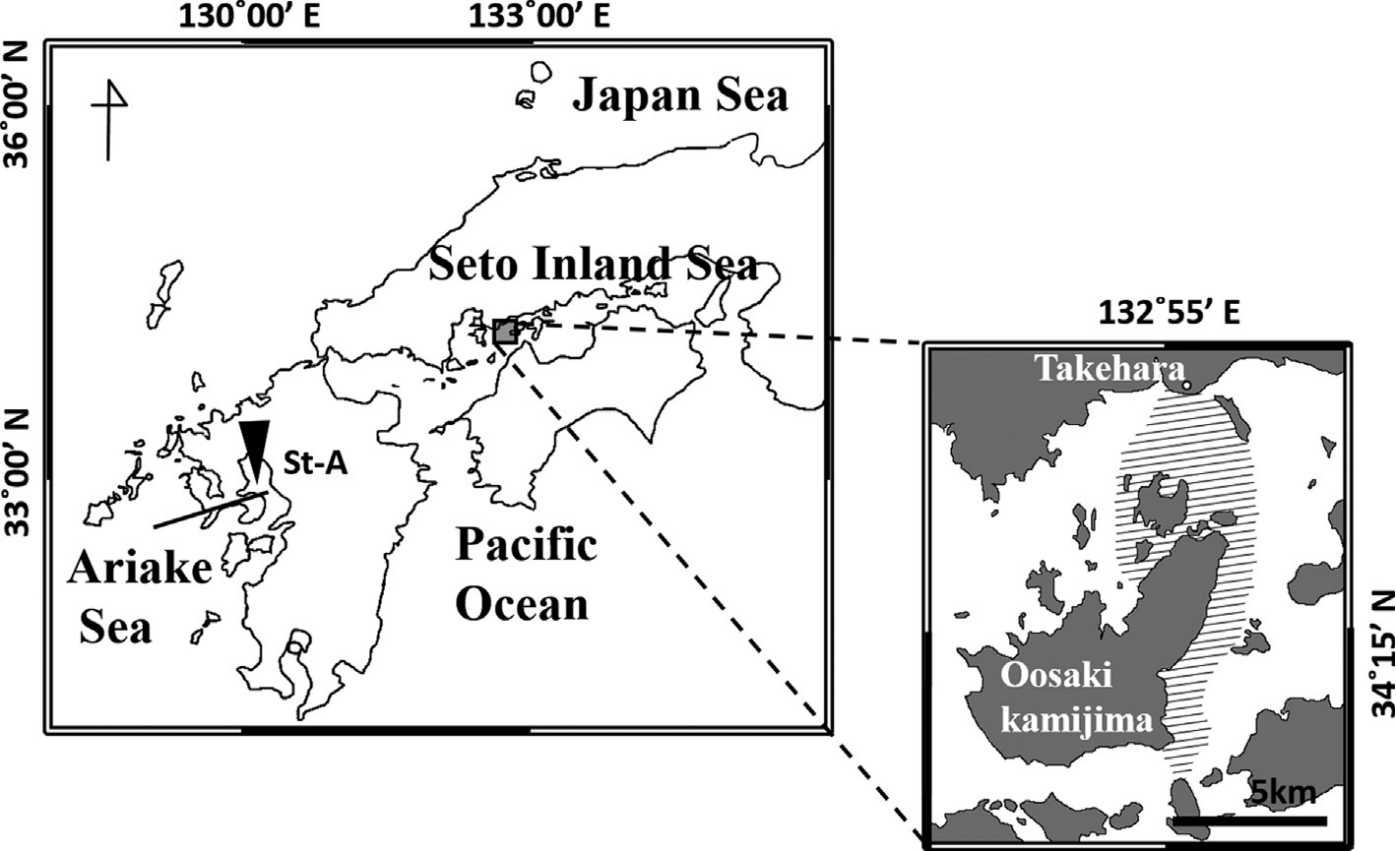
Sampling sites for jellyfish and juvenile fish in Japan. The shaded area indicates the sampling location in the Seto Inland Sea from March 2010 to September 2012. St-A indicates the supplementary sampling site in the Ariake Sea.

Scyphozoan jellyfish were collected with a scoop net (diameter 50 cm; mesh size 2 mm) from a fishing boat in the central part of the Seto Inland Sea of western Japan (34°10′17′′–34°19′27′′ N, 132°53′49′′–132°57′45′′ E) (shaded area inserted in Fig. 1) from March 2010 to September 2012. Collections were conducted at least twice a month throughout the study period. Surface water temperature and salinity at each sampling site were measured *in situ* using a CTD (D-400F, JFE Advantech Co., Ltd.). The bell diameter of each specimen of jellyfish was measured in the laboratory immediately after collection. The presence or absence of metacercariae in the mesoglea was examined under a compound stereomicroscope (SZ6045, Olympus). Bell measurements are presented as the mean ± standard deviation (SD).

Metacercariae found in jellyfish and juvenile fish, and adult trematodes found in the guts of associated juvenile fish were carefully removed from the host’s tissues with fine forceps, and then fixed in hot 10% formalin, stained with Mayer’s haemalum, dehydrated in an ethanol series, cleared in a xylene series, and mounted in Canada balsam. Trematodes were identified using Bartoli and Bray [4], Bray [6], Ozaki [40], and Yamaguti [54–57]. Body length measurements are presented as the mean ± SD.

Seasonal changes in prevalence and intensity of trematodes in each species of jellyfish examined were calculated following Bush et al. [9]. The mean intensity of trematodes in each of the host jellyfish throughout the study period was tested using the Steel-Dwass test because our data was not in normal distribution and homoscedasticity. Spearman’s rank correlation coefficient between host size (bell diameter and wet weight) and intensity of trematodes was tested. Statistical analyses were performed using R software (version 3.0.1).

### Examination of gut contents of juvenile fish

Juvenile fish associated with jellyfish were captured using a scoop net in the Seto Inland Sea from March 2010 to September 2012. Because juvenile fish were closely associated with their host jellyfish, they were easy to catch by scoop net. The juvenile fish were preserved in 99.5% ethanol soon after collection. The standard length (SL) of all juvenile fish specimens was measured and is presented as the mean ± SD. The gut contents of juvenile fish captured in the Seto Inland Sea were examined under a compound stereomicroscope (SZ6045, Olympus).

### Stable isotope analysis of jellyfish and their associated fish

Stable isotope analysis was used to evaluate the trophic interaction between a host jellyfish and associated juvenile fish. Juvenile fish and jellyfish were collected as described above from the Seto Inland Sea, and supplementary collection was made at a station in the Ariake Sea (St-A: 33°01′′25′′ N, 130°18′24′′ E) on October 2, 2011 (Fig. 1). The specimens were preserved in 99.5% ethanol. A marginal part of the bell of the jellyfish and part of the body muscles of the juvenile fish was cut off with clean scissors, dried at 60 °C in an oven (DOV-450P, AS ONE Co.), and pulverized using a mortar and pestle. The dried specimens were transferred to microtubes and subsequently treated with 1 mL methanol and 1 mL dichloromethane/methanol (7:1), and rinsed with distilled water to remove lipids [37]. In addition, carbonate carbon was removed from the specimens with 1 M HCl. The specimens were washed with distilled water and dried again prior to stable isotope analysis. Measurements of carbon and nitrogen isotope compositions were performed using an on-line system of ThermoFinnigan Delta Plus XP isotope-ratio mass spectrometry coupled with a Flash EA 1112 Automatic Elemental Analyzer through a ConFlo III interface modified to sensitive analysis [36]. Isotope compositions were expressed in conventional δ notation against the Peedee Belemnite (PDB) for carbon and atmospheric N_2_ for nitrogen:

$$\begin{array}{c} \delta^{13}C\;=\left({}^{13}R_{\mathrm{sample}}/{}^{13}R_{\mathrm{standard}}-1\right)\;\times 1000,\\ \delta^{13}N=\left({}^{15}R_{\mathrm{sample}}/{}^{15}R_{\mathrm{standard}}-1\right)\;\times 1000, \end{array}$$

where ^13^R and ^15^R are ^13^C/^12^C and ^15^N/^14^N ratios, respectively. Analytical errors (95% probability) were estimated to be within 0.3‰ for both carbon and nitrogen based on the repeated measurements of authentic and laboratory standards [36, 50].

## Results

### Identification of metacercariae in jellyfish

**Figure 2.**
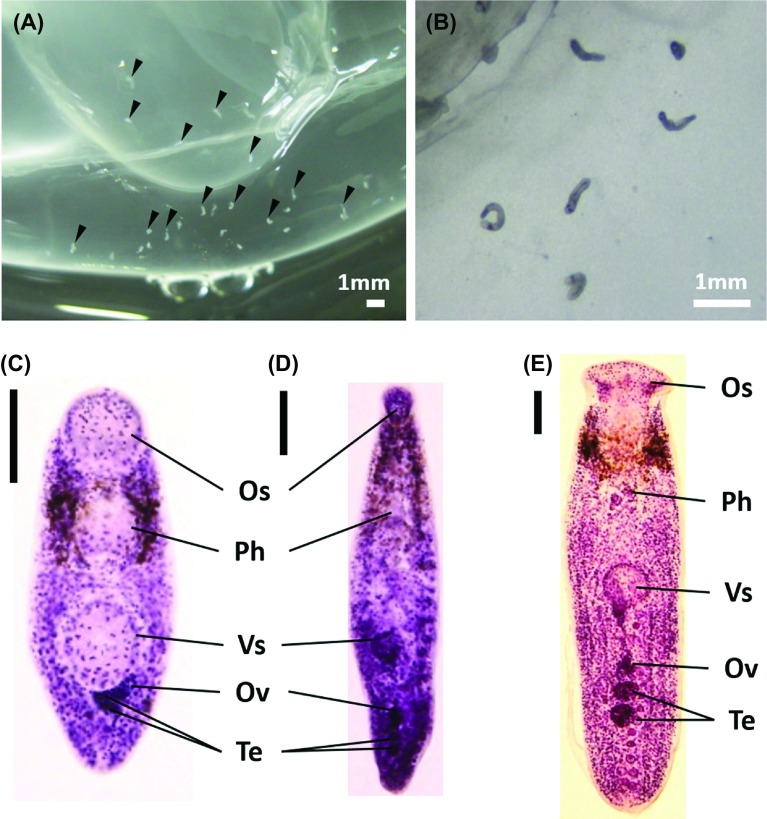
Metacercariae (indicated by black arrowheads) in the mesoglea of *Aurelia aurita* s.l. collected from the Seto Inland Sea (A, B). Stained metacercariae of *Lepotrema clavatum* (C), *Cephalolepidapedon saba* (D), and *Opechona olssoni* (E) (scale bars: 0.1 mm). Abbreviations: Os, oral sucker; Ph, pharynx; Vs, ventral sucker; Ov, ovary; Te, testis.

Large-sized scyphozoan jellyfish collected from the sampling area were identified as three species, *A. aurita* s.l., *C. pacifica*, and *C. nozakii*. All jellyfish collected were infected by unencysted metacercariae of three lepocreadiid trematode species, *L. clavatum*, *C. saba*, and *O. olssoni*, in the host mesoglea (Fig. 2). In total, 12,425 metacercariae of *L. clavatum* were obtained, which was greater than those of *C. saba* (*n* = 7,101) and *O. olssoni* (*n* = 3,463).

The morphology of the metacercariae is briefly described as follows: In *L. clavatum* (Fig. 2C), the body is pyriform, measuring 0.29–0.40 mm long (0.35 ± 0.03 mm, *n* = 18). This species is smallest among the three species. The tegument is spinose. The oral sucker (Os in Fig. 2C) and ventral sucker (Vs in Fig. 2C) are rounded. The pharynx (Ph in Fig. 2C) is oval. The ovary (Ov in Fig. 2C) and two testes (Te in Fig. 2C) are seen in the hindbody. The excretory vesicle is I-shaped, extending between the ventral sucker and intestinal bifurcation. The excretory pore terminal opens into dorsal side. In *C. saba* (Fig. 2D), the body is elongate, measuring 0.38–0.50 mm long (0.44 ± 0.05 mm, *n* = 19). The oral sucker (Os in Fig. 2D) is short and funnel-shaped. The ventral sucker (Vs in Fig. 2D) is rounded. The prepharynx is long. The pharynx (Ph in Fig. 2D) is oval. The intestines end blindly. The excretory vesicle extends to the pharynx. In addition, in *C. saba*, the circumoral spines are not seen clearly in the present metacercariae. The excretory vesicle reaches the pharynx in the present metacercariae. In this respect, our findings agree with the description by Yamaguti [56], but differ from those of Bartoli and Bray [4] and Shimazu [43, 44], who found that the excretory vesicle extends to the ovary or posterior testis. In *O. olssoni* (Fig. 2E), the body is elongate, measuring 0.69–1.15 mm long (0.92 ± 0.18 mm, *n* = 9), twice as large as the others. The oral sucker (Os in Fig. 2E) is elongate and funnel-shaped. The ventral sucker (Vs in Fig. 2E) is rounded and smaller than the oral sucker. The prepharynx is relatively short. The intestines and excretory vesicle form a uroproct (possibly a cloaca). The excretory vesicle extends slightly anterior to the intestinal bifurcation.

### Seasonal changes in prevalence and intensity of metacercariae in jellyfish

**Figure 3.**
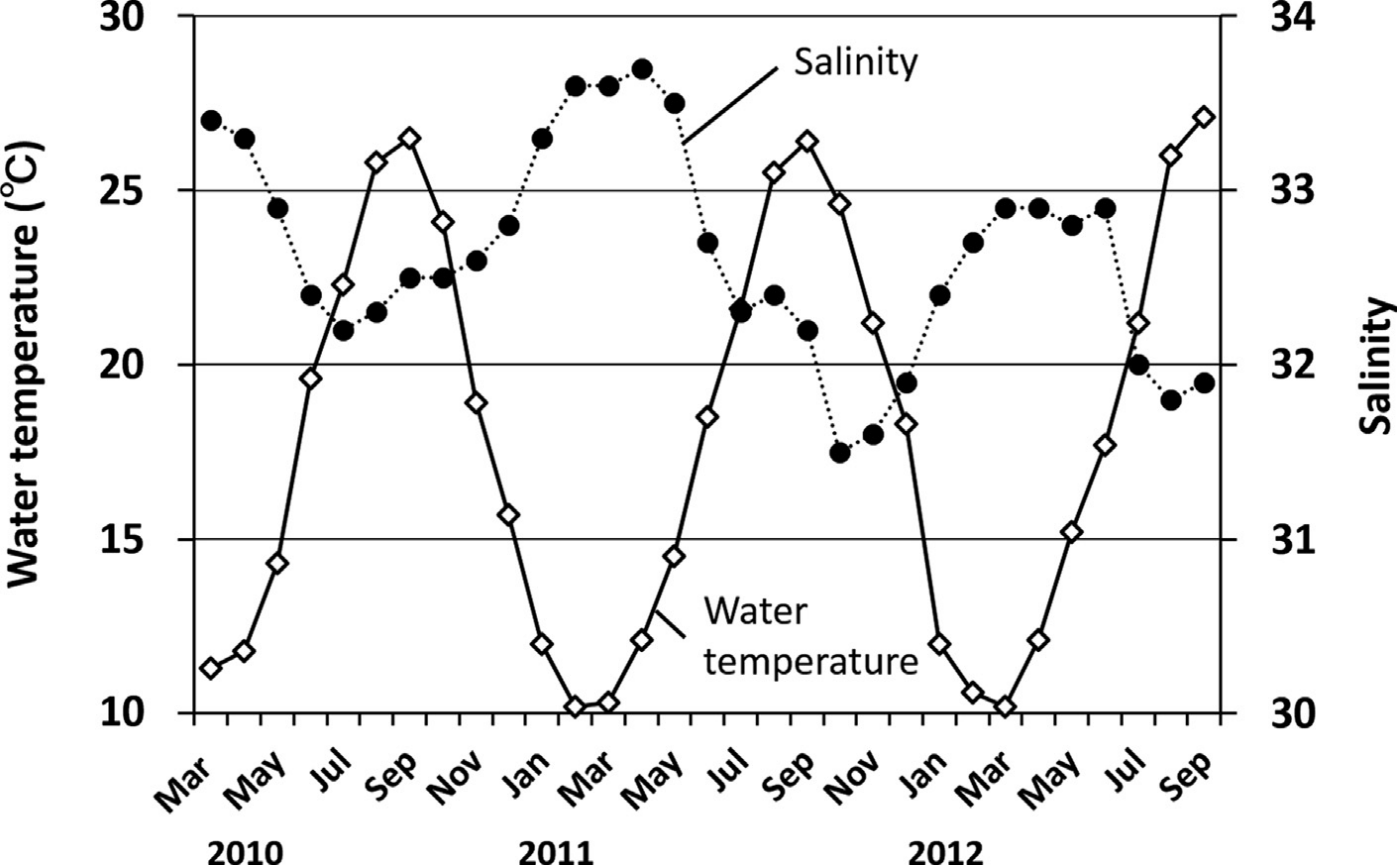
Seasonal changes in water temperature and salinity in the western part of the Seto Inland Sea from March 2010 to September 2012.

Seasonal changes in water temperature and salinity during the present investigation are shown in Figure 3. Water temperatures ranged from 10.2 °C (February 21, 2011) to 27.1 °C (September 18, 2012), and salinity fluctuated from 31.5 (October 12, 2011) to 33.7 (April 14, 2011).

**Figure 4.**
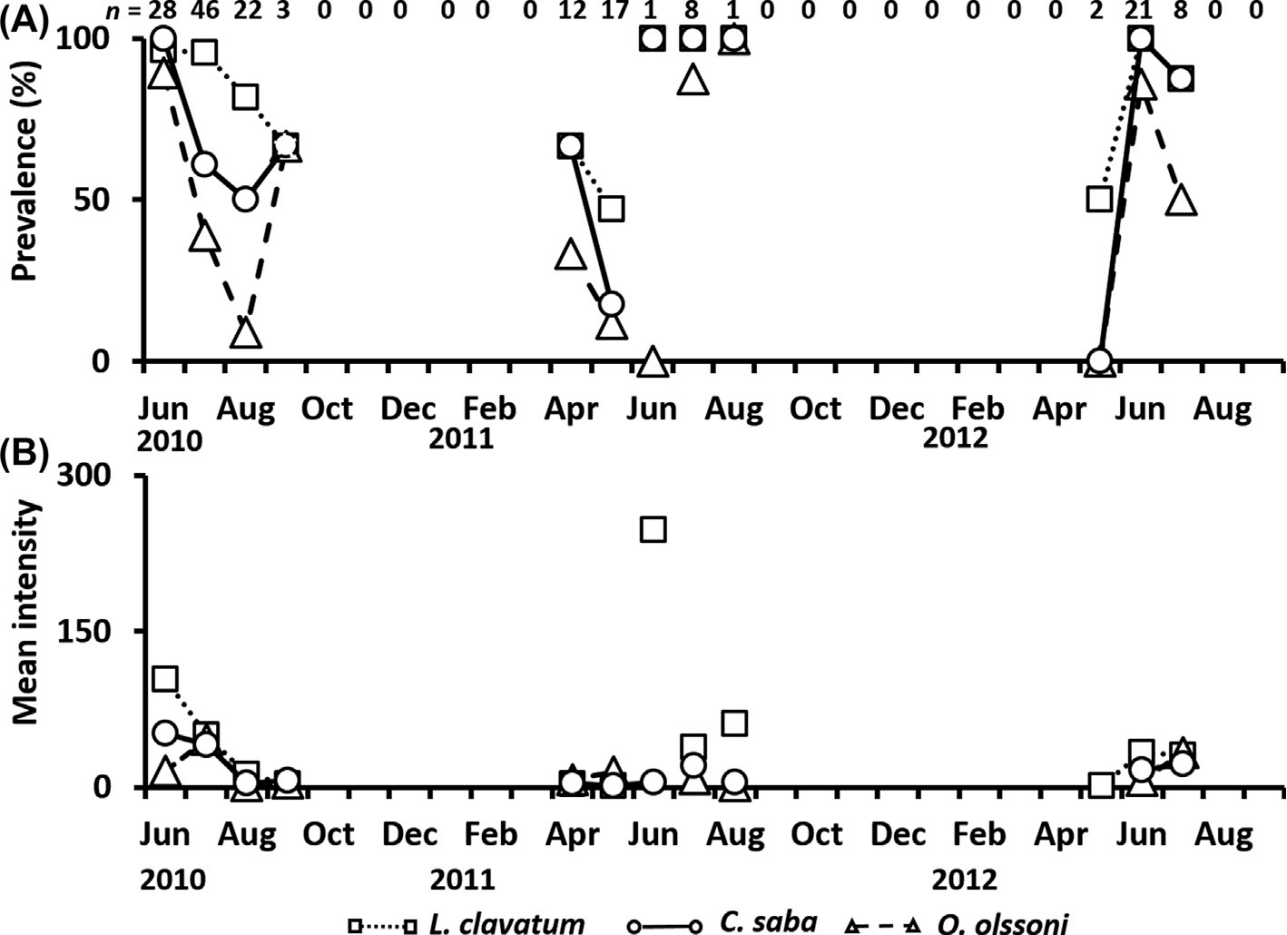
Seasonal changes in the prevalence (A) and mean intensity (B) of metacercariae in *Aurelia aurita* s.l. from June 2010 to September 2012. *Lepotrema clavatum*, open square; *Cephalolepidapedon saba*, open circle; *Opechona olssoni*, open triangle. *n*: Number of jellyfish collected. A line was not connected when only one individual was collected.

*Aurelia aurita* s.l. occurred from June to September in 2010, April to August in 2011, and May to July in 2012. A total of 174 individuals were investigated. The bell diameter ranged from 5.8 to 29.0 cm (13.3 ± 3.8 cm). Prevalence and mean intensity of metacercariae in *A. aurita* s.l. clearly showed seasonality (Figs. 4A and 4B), although the number of jellyfish collected varied widely among months. In *L. clavatum*, prevalence increased from March to June, and exceeded 95% in June of each year (Fig. 4A), excluding the months when only one individual was collected. The mean intensity of *L. clavatum* in *A. aurita* s.l. ranged widely from 1.0 to 247.0 individuals, and was consistently high in June of each year, decreasing thereafter (Fig. 4B). An extremely high intensity (440.0) was recorded on June 24, 2010. The mean intensities of *C. saba* and *O. olssoni* varied from 1.3 to 51.6 and from 0 to 45.6, respectively. They were much lower than that of *L. clavatum*, but generally exhibited a similar seasonal pattern (Fig. 4B).

**Figure 5.**
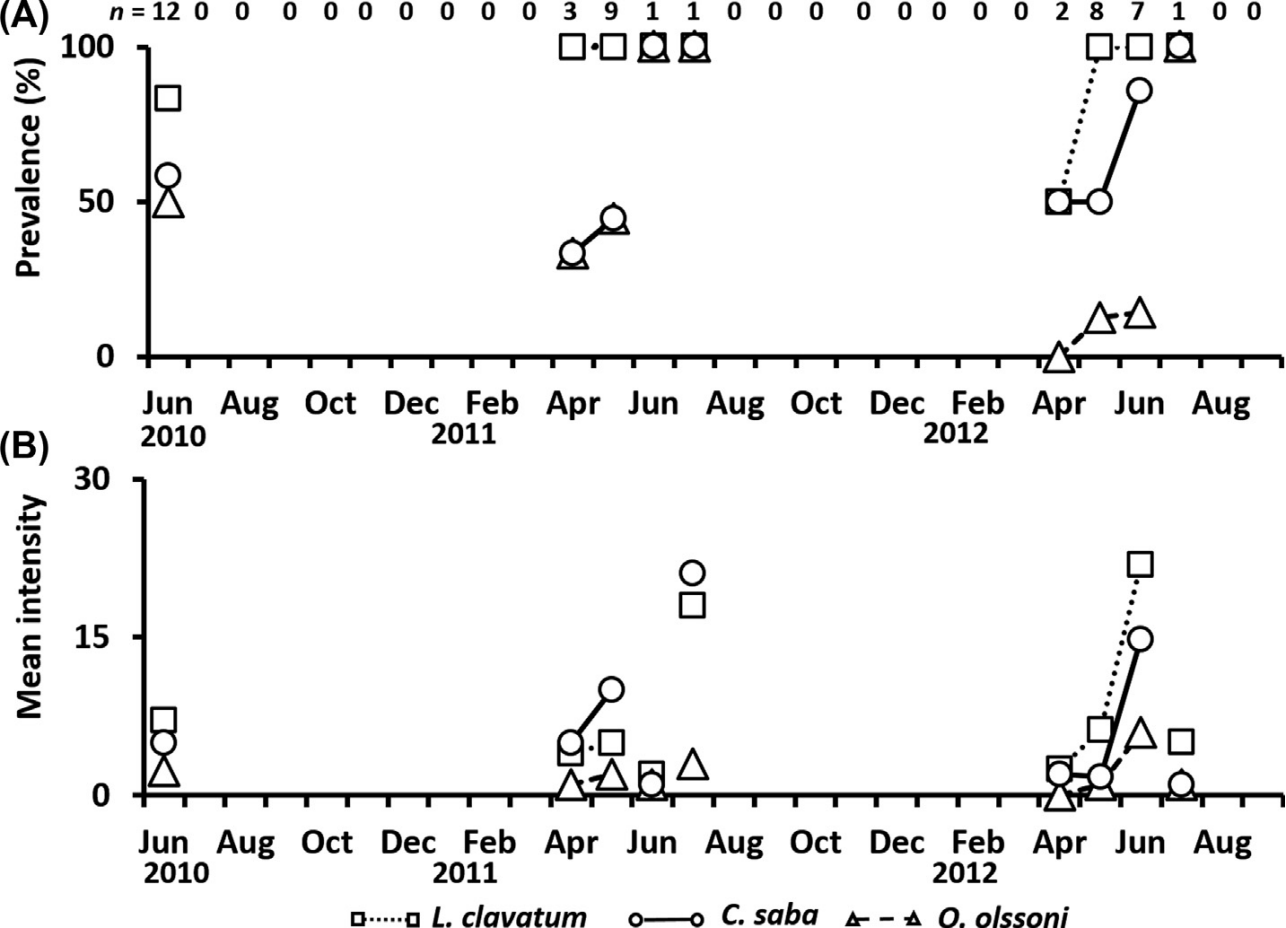
Seasonal changes in the prevalence (A) and mean intensity (B) of metacercariae in *Chrysaora pacifica* from June 2010 to September 2012. *Lepotrema clavatum*, open square; *Cephalolepidapedon saba*, open circle; *Opechona olssoni*, open triangle. *n*: Number of jellyfish collected. A line was not connected when only one individual was collected.

**Figure 6.**
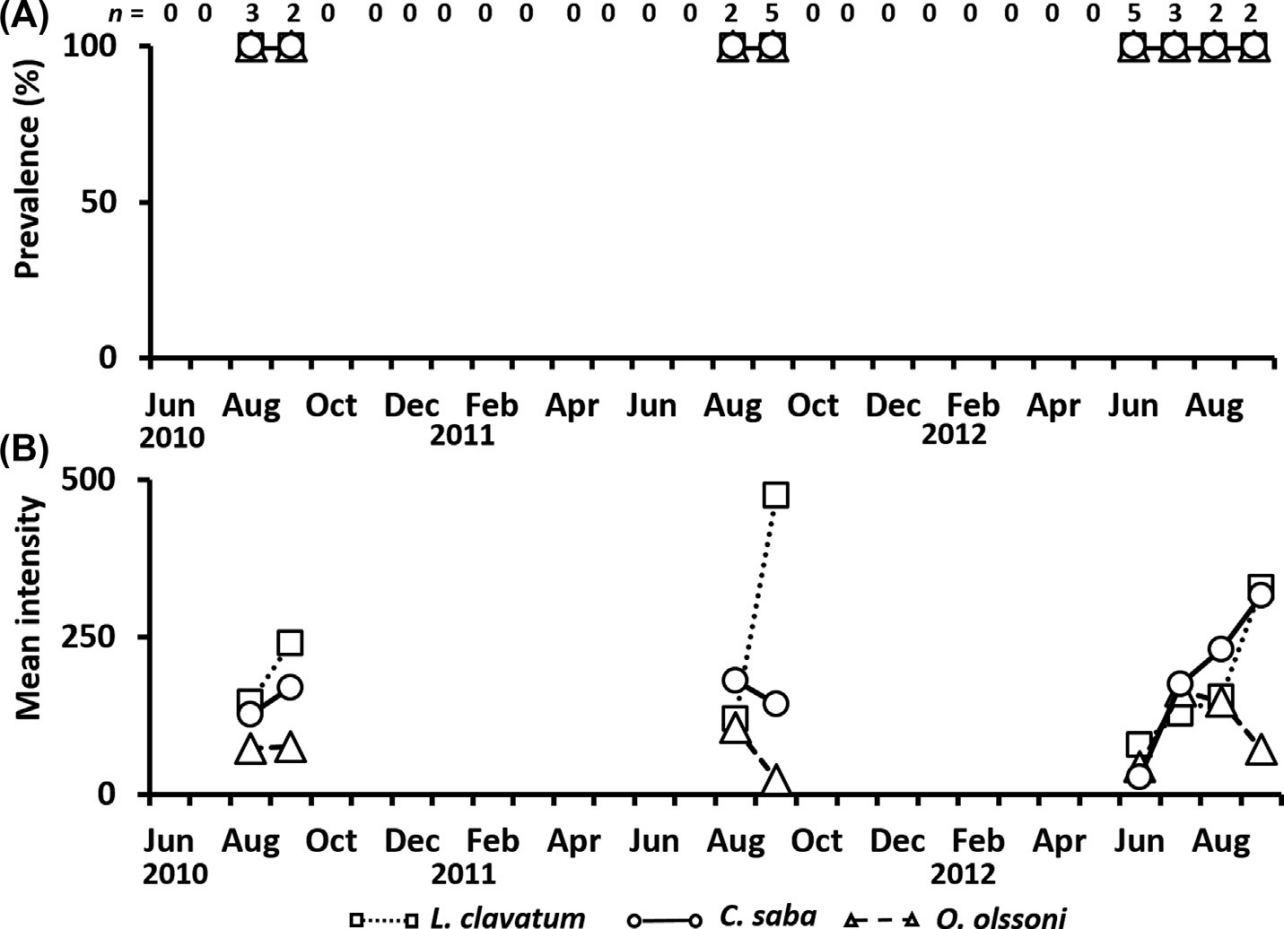
Seasonal changes in the prevalence (A) and mean intensity (B) of metacercariae in *Cyanea nozakii* from June 2010 to September 2012. *Lepotrema clavatum*, open square; *Cephalolepidapedon saba*, open circle; *Opechona olssoni*, open triangle. *n*: Number of jellyfish collected.

In *C*. *pacifica*, a total of 44 individuals were collected from June and April in 2010 to July in 2011 and 2012. The bell diameter ranged from 4.0 cm to 24.0 cm (14.2 ± 4.7 cm). The prevalence of *L. clavatum* was 50%–100% throughout the period of host occurrence (Fig. 5A). In *C. saba* and *O. olssoni*, the prevalence increased between April and July. The maximum mean intensity of the three trematode species was 21.9 in *L. clavatum* (Fig. 5B). The intensities of all species of trematodes were much lower than in *A. aurita* s.l. and *C. nozakii* (Figs. 4B, 5B, and 6B).

Twenty-four individuals of *C. nozakii* were sampled and examined from August and September in 2010 and 2011, and June to September in 2012. The bell diameter ranged widely from 13.3 cm to 51.0 cm (29.2 ± 10.1 cm). Prevalence of the three trematode species was consistently 100% during the period of the host’s occurrence (Fig. 6A), although it should be noted that replications of the sampled jellyfish were low (*n* = 2–5). The mean intensity of *L. clavatum* in *C. nozakii* ranged from 76.3 to 474.4 individuals. The mean intensities of *C. saba* and *O. olssoni* varied from 26.6 to 315.5 and 23.4 to 164.0, respectively (Fig. 6B).

**Figure 7.**
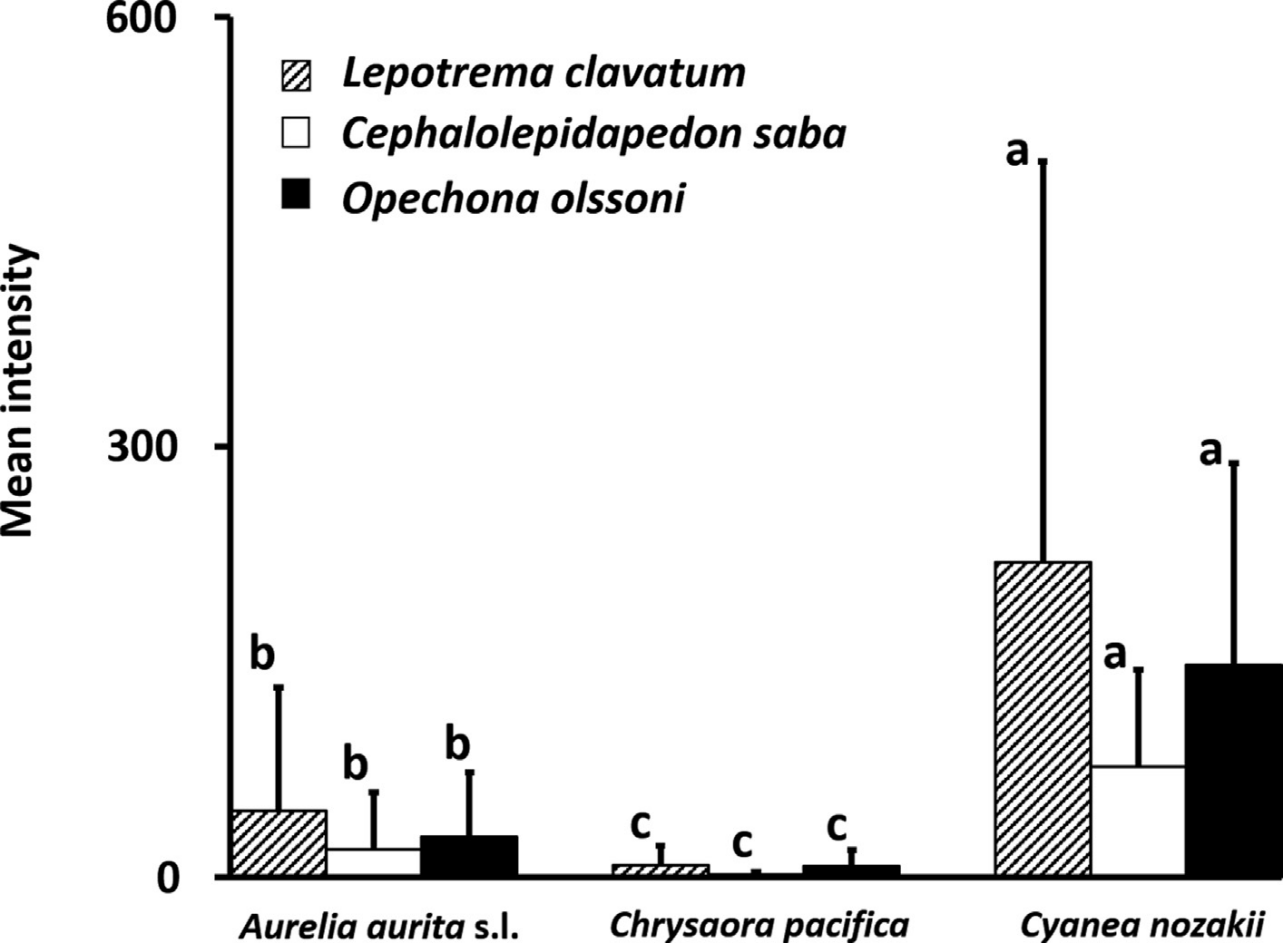
Mean intensity of metacercariae of each species in host jellyfish collected from June 2010 to September 2012. Error bars indicate standard deviations. Different letters denote significant differences among hosts (Steel-Dwass test, *p* < 0.05).

The mean intensity of *L. clavatum* in *C. nozakii* throughout the study period was 219.6. In *A. aurita* s.l. and *C. pacifica*, the mean intensities of *L. clavatum* were 46.5 and 8.8, respectively. There was a significant difference among the host jellyfish (Steel-Dwass test, *p* < 0.05). The mean intensity was highest in *C. nozakii* (147.8), compared to *A. aurita* s.l. (28.3) and *C. pacifica* (7.7). In *O. olssoni*, the mean intensity was 19.3 in *A. aurita* s.l., 2.2 in *C. pacifica*, and 77.0 in *C. nozakii.* In addition, significant differences in mean intensities among host jellyfish were observed for the other two trematodes (Steel-Dwass test, *p* < 0.05). The mean intensity of the three trematode species in *C. nozakii* was highest among the host jellyfish (Fig. 7).

**Figure 8.**
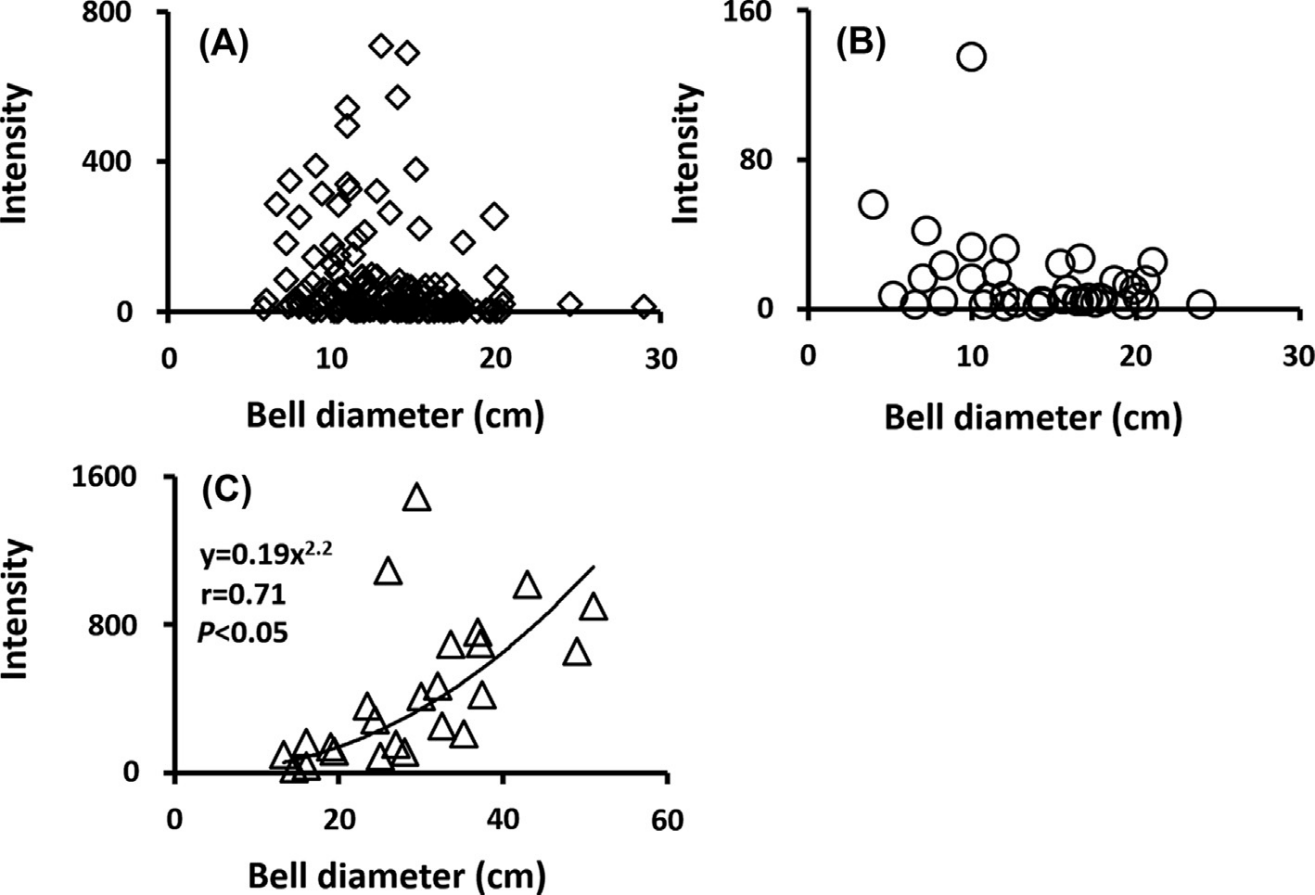
Relationship between bell diameter and intensity of all metacercariae. r: correlation coefficient. (A) *Aurelia aurita* s.l., (B) *Chrysaora pacifica,* (C) *Cyanea nozakii.*

Spearman’s rank correlation coefficient was calculated to examine any statistical correlation between the host’s bell diameter and the intensity of metacercariae (Fig. 8). Positive correlations between bell diameter and intensity of the metacercariae were significant only in *C. nozakii* (*r* = 0.71, *p* < 0.05) but not in the other two host species (*p* > 0.05). However, there was no correlation between wet weight and intensity (*p* > 0.05).

### Examination of the gut contents of juvenile fish

**Figure 9.**
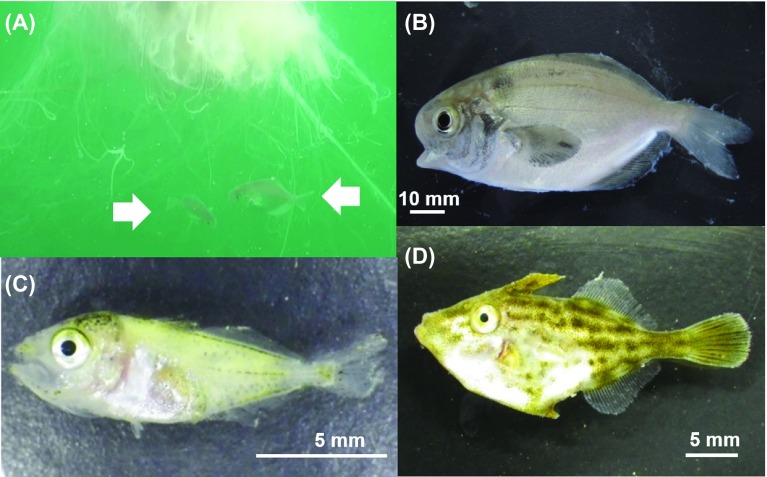
Juvenile fish associated with jellyfish collected from the Seto Inland Sea. (A) *Psenopsis anomala* associating with *Cyanea nozakii* (arrows). (B) *Psenopsis anomala.* (C) *Trachurus japonicus.* (D) *Thamnaconus modestus.*

The three species of jellyfish were accompanied by juvenile fish of *P. anomala, T. modestus*, and *T. japonicus* in the Seto Inland Sea (Fig. 9). *Psenopsis anomala* co-occurred with *C*. *pacifica* and *C*. *nozakii* from May to September. *Thamnaconus modestus* and *T. japonicus* were associated with *A. aurita* s.l. and *C. nozakii* from June to August (Table 1). *Psenopsis anomala* also co-occurred with *C*. *nozakii* in the Ariake Sea.

**Table 1.** Number and size of fish juveniles associated with jellyfish in Seto Inland Sea from July 2010 to September 2012. Abbreviations: SL, standard length of associated fish (cm); SD, standard deviation.

Associated fish	Date	Total number	SL (cm) (mean ± SD)	Host jellyfish	Total number	Prevalence (%)
*Psenopsis anomala*	August 2010	9	5.3 ± 2.4	*Cyanea nozakii*	11	54.5
	September 2010	1	7.4	*Cyanea nozakii*	10	10.0
	May 2011	1	2.2	*Chrysaora pacifica*	3	11.1
	June 2011	1	4.3	*Chrysaora pacifica*	9	100
	July 2012	2	5.7 ± 3.5	*Cyanea nozakii*	3	33.3
	August 2012	4	7.3 ± 3.3	*Cyanea nozakii*	8	37.5
*Trachurus japonicus*	July 2010	8	1.0 ± 0.3	*Aurelia aurita* s.l.	46	4.3
	August 2010	1	1.5	*Cyanea nozakii*	11	9.1
	June 2012	2	1.3 ± 0.3	*Aurelia aurita* s.l.	21	4.8
	June 2012	25	0.9 ± 0.1	*Cyanea nozakii*	5	40.0
	July 2012	2	2.4 ± 0.3	*Cyanea nozakii*	3	33.3
	August 2012	121	1.0 ± 0.2	*Cyanea nozakii*	8	37.5
*Thamnaconus modestus*	June 2012	1	2.5	*Aurelia aurita* s.l.	21	4.8
	June 2012	1	2.6	*Cyanea nozakii*	3	33.3
	July 2012	2	2.3 ± 0.3	*Aurelia aurita* s.l.	8	25.0

**Figure 10.**
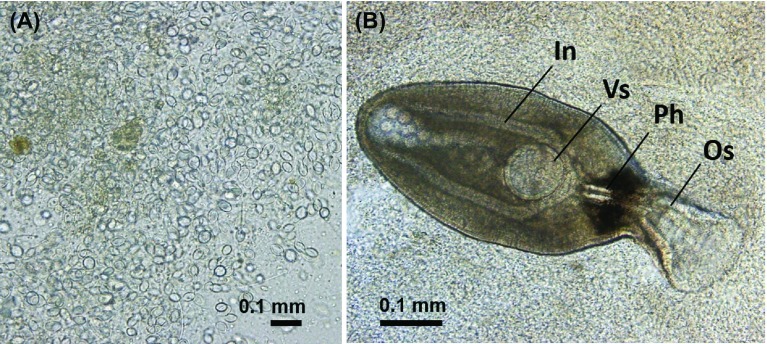
Nematocysts (A) and metacercaria of *Opechona olssoni* (B) observed in the gut of a juvenile butterfish, *Psenopsis anomala* (standard length: 6.6 cm), collected in August 27, 2010. Abbreviations: Os, oral sucker; Ph, pharynx; Vs, ventral sucker; In, intestine.

Numerous nematocysts of unidentified cnidarians were observed in the guts of *P. anomala* (SL: range 2.2–9.7 cm; mean ± SD 5.4 ± 2.2 cm; *n* = 11) and *T. modestus* (SL: 2.0–2.9 cm; 2.5 ± 0.4 cm; *n* = 4), implying that *P. anomala* and *T. modestus* had fed on jellyfish. In addition, metacercariae and adults of *O. olssoni* and *C. saba,* and metacercariae of *L. clavatum* were detected in the guts of *P. anomala*. Metacercariae of *L. clavatum* and *O. olssoni* were also detected in the guts of *T. modestus* (Table 2, Fig. 10). In contrast, neither trematodes nor nematocysts were observed in the guts of *T. japonicus* (SL: 1.0–2.7 cm; 1.8 ± 0.7 cm; *n* = 5) (Table 2).

**Table 2.** Occurrence of trematodes in fish juveniles associated with jellyfish in Seto Inland Sea from August 2010 to July 2012. Abbreviations: No, Number of examined fish juveniles; +, Nematocysts are contained in guts; −, Nematocysts are not contained in guts; Pm, Prevalence of metacercariae (%); Im, Mean intensity of metacercariae; Pa, Prevalence of adults (%); Ia, Mean intensity of adults.

Host fish	No	Nematocysts (%)	Trematodes											
			*Lepotrema clavatum*				*Cephalolepidapedon saba*				*Opechona olssoni*			
			Pm	Im	Pa	Ia	Pm	Im	Pa	Ia	Pm	Im	Pa	Ia
*Psenopsis anomala*	11	+ (100)	18.2	1	0	0	27.3	2.3	54.5	.2.7	63.6	4.6	18.2	1
*Trachurus japonicus*	4	− (0)	0	0	0	0	0	0	0	0	0	0	0	0
*Thamnaconus modestus*	4	+ (100)	100	2.3	0	0	0	0	0	0	50	1	0	0

### Stable isotope analysis of jellyfish and their associated fish

**Figure 11.**
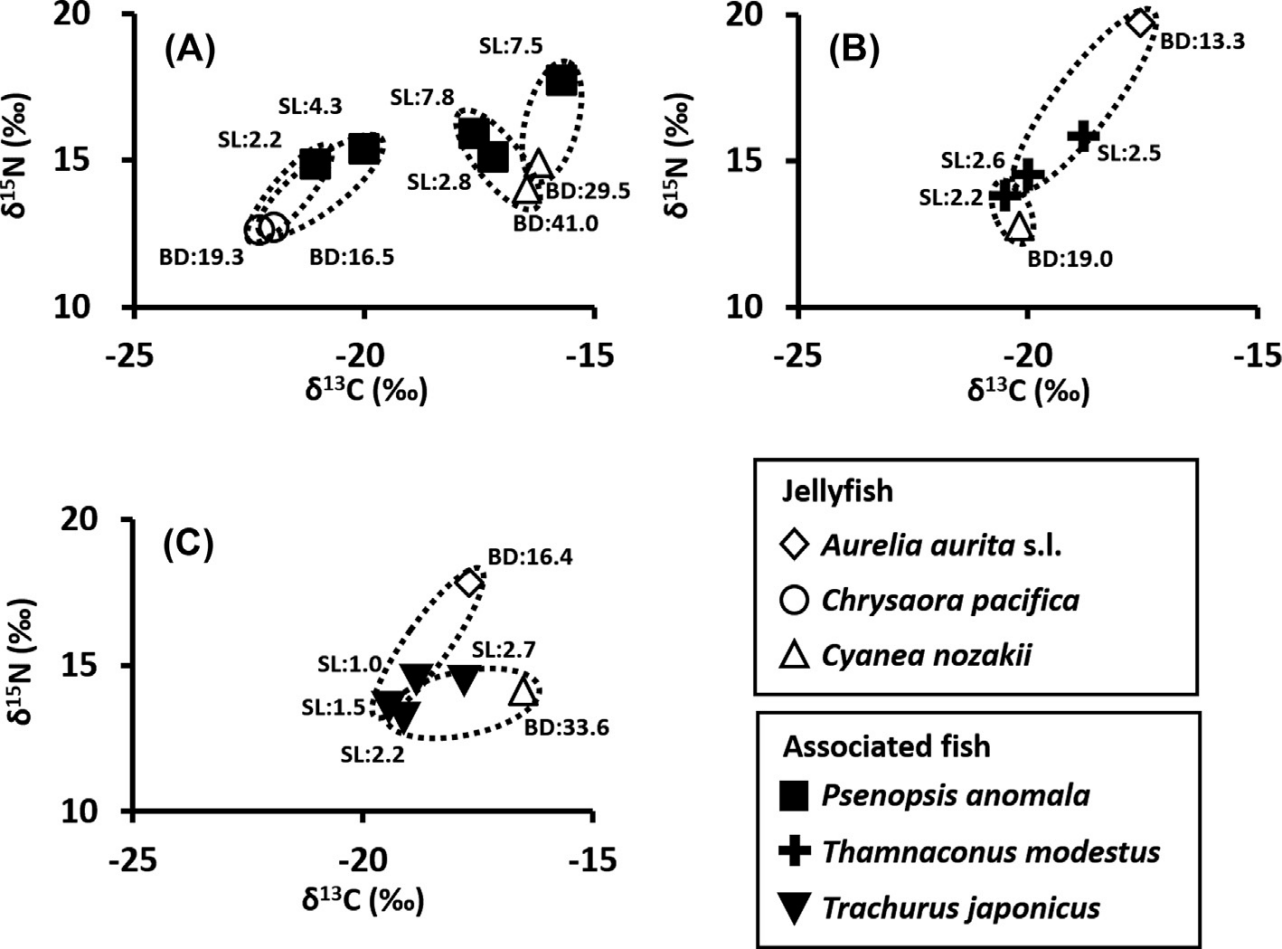
Dual isotope plot of δ^13^C and δ^15^N. (A) *Psenopsis anomala* and its host jellyfish, (B) *Thamnaconus modestus* and its host jellyfish, (C) *Trachurus japonicus* and its host jellyfish. Dotted lines indicate pairs of an associated fish and its host jellyfish which were collected together. Abbreviations: BD, bell diameter of host jellyfish (cm); SL, standard length of associated fish (cm).

The δ^13^C values of *A. aurita* s.l. (*n* = 3) ranged from −17.7‰ to −17.5‰. The δ^15^ N values were 17.8‰–19.7‰ (Table 3). In *C. pacifica* (*n* = 2), δ^13^C values and δ^15^N values were −22.3‰, 12.6‰ and −22.0‰, 12.7‰, respectively. The δ^13^C values ranged from −20.2‰ to −16.5‰, and δ^15^N values ranged from 12.8‰ to 14.1‰ in *C. nozakii* (*n* = 4). Each jellyfish and juvenile fish pair showed a different tendency. The δ^13^C values of *P. anomala* (*n* = 5) ranged from −21.2‰ to −17.2‰ and these values depended on their host jellyfish. Moreover, δ^15^N values (14.9‰ to 15.9‰) were higher than each host (Fig. 11). In pairs of *P. anomala* and *C. pacifica*, the mean δ^15^N value of *P. anomala* was 2.3‰ and 2.7‰ higher than those of their hosts, respectively. In contrast, the δ^13^C and δ^15^N values of *T. modestus* (*n* = 3) were similar among three individuals (−20.4‰ to −18.8‰ and 13.8‰ to 15.8‰). *Trachurus japonicus* also showed that the δ^13^C (−19.4‰ to −17.8‰) and δ^15^N values (13.2‰ to 14.6‰) were similar among the individuals examined. The δ^13^C and δ^15^N values of these two species were different from those of the host jellyfish.

**Table 3.** δ^13^C (‰) and δ^15^N (‰) contents of members of associations between jellyfish and fish. Abbreviations: BD, bell diameter of host jellyfish (cm); SL, standard length of associated fish (cm).

Host jellyfish	Locality	BD (cm)	δ^13^C (‰)	δ^15^N (‰)
*Aurelia aurita* s.l.	Seto Inland Sea	16.4	−17.5	17.8
	Seto Inland Sea	13.3	−17.6	19.7
	Seto Inland Sea	21.0	−17.7	17.8
*Chrysaora pacifica*	Seto Inland Sea	19.3	−22.3	12.6
	Seto Inland Sea	16.5	−22.0	12.7
*Cyanea nozakii*	Seto Inland Sea	19.0	−20.2	12.8
	Seto Inland Sea	33.6	−16.5	14.1
	Seto Inland Sea	41.0	−16.5	14.0
	Ariake Sea	29.5	−16.2	14.9
Associated fish	Locality	SL (cm)	δ^13^C (‰)	δ^15^N (‰)
*Psenopsis anomala*	Seto Inland Sea	2.2	−21.1	14.9
	Seto Inland Sea	4.3	−20.0	15.4
	Seto Inland Sea	2.8	−17.2	15.1
	Seto Inland Sea	7.8	−17.6	15.9
	Ariake Sea	7.5	−15.7	17.7
*Thamnaconus modestus*	Seto Inland Sea	2.6	−20.0	14.4
	Seto Inland Sea	2.5	−18.8	15.8
	Seto Inland Sea	2.2	−20.4	13.8
*Trachurus japonicus*	Seto Inland Sea	1.0	−18.8	14.6
	Seto Inland Sea	1.5	−19.4	13.6
	Seto Inland Sea	2.2	−19.1	13.2
	Seto Inland Sea	2.7	−17.8	14.5

## Discussion

### Jellyfish as intermediate and/or paratenic hosts of metacercariae

In the present study, all individuals of the trematodes observed in jellyfish were identified as unencysted metacercariae belonging to the family Lepocreadiidae. No specific information regarding the first intermediate hosts of these trematodes is available. However, sporocysts full of oculate trichocercous cercariae (Lepocreadiidae) in the dove shell, *Mitrella bicincta* (Gould, 1860) from the Seto Inland Sea in May (Kondo, unpublished data), may be a candidate for one of the three trematodes. In the present study, infection of jellyfish by metacercariae showed distinct seasonal differences in prevalence and mean intensity in the Seto Inland Sea. Martell-Hernández et al. [26] reported the seasonal occurrence of *Opechona pyriforme* (Linton, 1900) metacercariae in the hydrozoan *Eirene tenuis* (Browne, 1905) in the western Gulf of Mexico. The host occurred in May, July, and October, whereas the endoparasite was observed in the mesoglea of the host only in July and October, suggesting that the infectious period of the parasite to the host was restricted to July and October. In addition, Yip [58] reported that the prevalence and intensity of *Opechona bacillaris* (Molin, 1859) metacercariae in the ctenophore *Pleurobrachia pileus* (O. F. Müller, 1776) were highest in Galway Bay, western Ireland, in early summer. These seasonal changes in prevalence and intensity are related to the life cycle of the trematodes. The trematode *Bucephalus* sp., which utilizes the Japanese pearl oyster *Pinctada fucata martensii* (Dunker, 1872) as its first intermediate host, actively releases free-swimming cercariae as water temperature increases [34]. In this study, the prevalence of metacercariae of three species in *A. aurita* s.l. and *C. pacifica* increased with a rise in water temperature from early spring to early summer (Figs. 3–5). Seasonal changes in prevalence and intensity in the second intermediate host seem to be controlled by the seasonal release of infective cercariae related to changes in water temperature.

In *C. nozakii*, which occurred from July to September, the prevalence and mean intensity of metacercariae were highest among the jellyfish examined in the present study, although the low samples sizes should be noted (Figs. 6 and 7). *Cyanea nozakii* is known as a medusivore [21]. Metacercariae of the family Lepocreadiidae have been reported to infect many species of gelatinous zooplankters, including ctenophores, hydrozoans, and scyphozoans [8, 33, 35]. In the Seto Inland Sea, *C. nozakii* has been observed feeding on ctenophores, hydrozoans, and scyphozoans (Kondo, personal observation). Therefore, the high prevalence and intensity of metacercariae on *C. nozakii* may be partly attributable to medusivory. Such an accumulation of metacercariae in paratenic hosts is known in other trematodes. In the trematode *Pharyngostomum cordatum* (Diesing, 1850) Ciurea, 1922, the intermediate, definitive, and paratenic hosts are frogs, cats, and snakes, respectively; in paratenic frog-eating hosts, metacercariae accumulate [14]. Moreover, a positive relationship was observed between bell diameter and intensity only in *C. nozakii* (Fig. 8C). Generally, it is not unusual for parasites to occur in greater abundance in larger hosts [22]. However, *A. aurita* s.l. and *C. pacifica* did not show significant positive relationships (Figs. 8A and 8B). It appears that accumulation may be due to medusivory by *C. nozakii*. Hansson [16] reported that predation by the lion’s mane jellyfish *Cyanea capillata* (Linnaeus, 1758) on *A. aurita* is enhanced as the bell diameter of the predator increases. In addition, such a tendency was also detected in the medusivorous scyphozoan fried egg jellyfish *Phacellophora camtschatica* (Brandt, 1835) [46]. Metacercariae are accumulated by predation of larger *C. nozakii* because large medusivorous jellyfish can catch a higher number of gelatinous zooplankters than small ones. Thus, it is presumed that *C. nozakii* plays a role as a paratenic host rather than a second intermediate host.

### Transmission of trematodes to definitive host fish via predation of infected jellyfish

In Japanese waters, it has been previously found that *T. modestus*, the thread-sail filefish *Stephanolepis cirrhifer* (Temminck and Schlegel, 1850), the lagoon triggerfish *Rhinecanthus aculeatus* (Linnaeus, 1758), and the fivespot flounder *Pseudorhombus cinnamoneus* Günther, 1862 are definitive hosts for *L. clavatum* [12, 39, 40, 54]; *P. anomala* and *T. modestus* are hosts for *O. olssoni* [39], and *P. anomala* and the chub mackerel *Scomber japonicus* Houttuyn, 1782, are hosts for *C. saba* [39, 44]. The definitive hosts of trematodes that use jellyfish as a second intermediate host have been summarized in previous studies [8, 35]. Infection from the intermediate or paratenic host jellyfish to the definitive host fish most likely occurs via predation by the definitive host [25, present study]. In the present study, three species of juvenile fish, *P. anomala*, *T. modestus,* and *T. japonicus* were collected along with associated jellyfish throughout the investigation (Table 1). Juveniles of these fish are known to be closely associated with jellyfish [17, 20, 24, 27, 29, 30, 38, 39, 41, 51, 52]. It seems that transmission of the metacercariae in the second intermediate and/or paratenic host to the definitive host also occurred via predation by the associated juvenile fish of infected jellyfish in the Seto Inland Sea.

Juveniles of *P. anomala* were observed swimming around the tentacles of jellyfish from May to September (Fig. 9A). These young fish are considered to utilize jellyfish as shelter to avoid predation by visual predators [5, 42]. According to Shojima [45], when reaching a total length of 1.5–2.0 cm, the larvae become juveniles. In the present study, the smallest individual (2.2 cm in standard length) was captured in May 2011. Therefore, it is likely that juveniles of *P. anomala* became associated with jellyfish before or immediately after metamorphosis. *Psenopsis anomala* is a medusivorous fish, as their guts often contain pieces of jellyfish gonads and tissues even as larvae and juveniles [45]. In the present study, nematocysts were frequently found in the guts of juvenile *P. anomala*. In addition, our stable isotope analysis also supported that juvenile *P. anomala* fed on the host jellyfish. Stable isotope ratios generally reflect dietary diversity. When the trophic level rises by one step in the food chain, δ^13^C and δ^15^N values increase by about 1‰ and 3‰, respectively [11, 31]. Because stable isotope ratios consistently increased, we can infer trophic relationships between prey and their predators. Recently, stable isotope analyses were used to reveal relationships between jellyfish and their symbionts [13, 53]. D’Ambra et al. [10] obtained evidence with this technique that associated juvenile fish *Chloroscombrus chrysurus* (Linnaeus, 1766) fed on their hosts jellyfish *Aurelia* sp. and *Drymonema larsoni* Bayha and Dawson, 2010. *Psenopsis anomala* was enriched in δ^15^N relative to host jellyfish in all pairs (see Table 3, Fig. 11). Furthermore, metacercariae and adults of trematodes were observed in the guts (Table 2), suggesting that these trematodes infected *P. anomara* via predation.

*Thamnaconus modestus* has previously been reported as the definitive host of *L. clavatum* [54]. In this study, the transmission of trematodes is presumed to have been caused through a prey-predator relationship between jellyfish and juveniles of *T. modestus*, because the nematocysts and metacercariae of trematodes were found together in the guts of the juveniles (Table 2). However, the δ^15^N value of *T. modestus* is not suggestive of predation by juvenile *T. modestus* on jellyfish (Fig. 11). Kim et al. [18] reported that most of the prey items of *T. modestus* belonged to four groups: hyperiid amphipods, gastropods, ophiuroids, and algae. Minagawa and Yoshioka [32] suggested that when a predator is highly omnivorous, its stable isotope values are estimated to be lower than expected, because they are equalized by the stable isotope values of various food items. Thus the stable isotope analysis suggests *T. modestus* feeds on jellyfish in addition to other foods, although it is unknown how much each prey item contributed to the results of the stable isotope analysis.

In contrast, the mean δ^15^N values of *T. japonicus* were nearly equal to or lower than that of the host jellyfish. The δ^13^C values of *T. japonicus* were less than that of the host jellyfish (Table 3, Fig. 11). In *T. japonicus*, neither nematocysts nor trematodes were detected in the guts (Table 2). Masuda [29] reported that *T. japonicus* measuring 1.0–2.7 cm in length used jellyfish for shelter, but not for food. The *T. japonicus* specimens collected in this study fall within the size range recorded by Masuda [29] (Table 1). Masuda [29] suggested that jellyfish function as shelter, rather than as a food source for juveniles of *T. japonicus.* Therefore, the gut contents and stable isotope analyses indicated no predation by *T. japonicus* juveniles on jellyfish. Although Machida et al. [23] reported that *Opechona* sp. was found in the pyloric cecum and intestines of *T. japonicus,* these fish, which were landed by commercial fishing boats, were adults. All *T. japonicus* collected in this study were juveniles. Therefore it seems unlikely that juvenile *T. japonicus* act as a definitive host for trematodes that infect jellyfish.

The present study clearly revealed that transmission of trematodes, from intermediate and paratenic hosts to definitive hosts, occurred over an approximately six-month period, using a wide variety of host jellyfish. The life cycles of the three trematode species examined in the present study were clarified, except for the first intermediate hosts. It seems likely that *C. nozakii* may act as paratenic hosts through medusivory. The following questions should be investigated in future studies: (1) When are miracidium larvae released from definitive hosts? (2) How do cercariae infect from first intermediate to second intermediate hosts? (3) How long can metacercariae survive in the host jellyfish? and (4) How long do adult trematodes survive in definitive hosts?
